# Individual freedom versus collective responsibility: an ethicist's perspective

**DOI:** 10.1186/1742-7622-3-11

**Published:** 2006-09-25

**Authors:** Richard E Ashcroft

**Affiliations:** 1 Institute of Health Sciences Education, Barts and the London School of Medicine and Dentistry, Queen Mary, University of London, UK

## Abstract

Philosophical theories of collective action have produced a number of alternative accounts of the rationality and morality of self-interest and altruism. These have obvious applications to communicable disease control, the avoidance of antibiotic resistance, the responsibility of healthcare professionals to patients with serious communicable diseases, and the sharing of personal data in epidemiological research.

There is no problem more central ethics than the question ''Why should I be moral?'' An influential way of posing this question is to put this in terms of self-interest. Why should I act in a way which is, or seems to me to be, contrary to my own personal interests, even where this may produce benefits, or avoid harm, to another person or group? There are a number of different theories of ethics which attempt to answer this question.

One popular approach is to draw on evolutionary theory and the theory of games. In this approach, it is argued that although self-interested behaviour may pay off in the short term, in the long term each individual benefits by cooperating (including occasional sacrifices of one's own interests for the benefit of others). This approach has been elaborated in evolutionary theory and moral philosophy by many writers[1-3]. The appeal of this theory is quite broad. Firstly, it seems to be empirically grounded. It is not a pious appeal to a principle such as "Do as you would be done by", but a demonstrably successful evolutionarily stable strategy. Second, it seems to prove that there is no radical distinction between morality and self-interest. Morality is just a generalised version of self-interest. The genuinely self-interested person would be moral, because this is the strategy with the best long-term expected pay-off for him or her. Trade-offs of his or her self-interest are at worst short-term losses that can be considered investments for long-term gain.

This argument is, moreover, appealing in practice. It gives a plausible account of why parents ought to vaccinate their children against communicable diseases, why people with sexually transmitted diseases should be encouraged to tell their partners (even where telling might risk the continuity of a desired relationship), and why people ought not to take antibacterials for viral diseases and ought to comply with medical instructions when taking antibiotics.

However, a lot of this appeal is only apparent. The game theoretic argument usually founders, for three reasons. Firstly, in many cases in modern societies it is hard to persuade people to sacrifice their interest to unknown strangers, and there is often a strong common interest in not doing so (for instance in cooperating with one's own declared enemies over some public health problem). Secondly, in many cases there is a short, quite definite series of interactions, so that the pay-off for cheating may in fact be strong. For instance, each parent needs to choose to vaccinate each child only once, so non-vaccination looks like a rational strategy if *enough *other parents vaccinate. This strategy is an instance of the well known "free-rider" problem in economic theory. Thirdly, the model depends on there being agreement about what the costs, risks and pay-offs really are. In the measles-mumps-rubella (MMR) vaccine case, some parents may disagree with the healthcare professionals not only about the degree of risk posed by the vaccine but also about the seriousness of autism vis-à-vis measles (say), and hence calculate their pay-off differently. Under these circumstances, what looks like a rational trade-off to the professional looks irrational to the parent purely because the calculations of benefit and harm differ.

The problems identified here underlie many interesting issues in public health policy, but in ethical theory the most interesting point is that if morality rests on an extended theory of self-interest alone, then any specific proposal about collective action can founder on the three problems I have discussed here. If we want people to act morally, it is not because so acting is in enlightened self-interest alone, but because sometimes we need people to make genuine sacrifices of their interests to the benefit of others: to act against their interests, in the certainty or high probability of personal loss. Although evolutionary theory does suggest that sacrifice by individuals in a species can be explained by benefit to other individuals with common genetic material, I suggest that this approach does not entirely circumvent the issue, and we need to consider a properly moral theory rather than a rational interest theory [4].

What should such a theory look like? Most recent work in the ethics of public health applies a utilitarian, a communitarian, or a liberal approach. For a utilitarian, we ought to promote the greatest good for the greatest number of people. Doing so may involve coercive measures which compel individuals to act against their own perceived best interests in the name of the public good. Coercive policies have the obvious disadvantages, first, that they may create *improper *infringements of individual rights, and, second, that they may grant too great an authority to specific individuals or agencies to determine of what the public good consists[5]. For a communitarian, there is a similar emphasis on the public good, combined with the idea that our personal interests and identities are formed out of our membership of a community with specific values, rather than the nature of the community being fixed by our collected individual interests. Again, appeal to common interests solves the problem of individual-collective conflicts, but arguably at too high a cost in terms of infringements of individual liberties[6].

The most likely solutions lie within liberalism. Following the ideas of John Stuart Mill, liberals believe that individuals should be free to live as they think best, subject only to the limitation that their actions and choices should not cause harm to others. This captures the idea that we should respect individual rights but also identify strict limits to those rights. A difficulty is that it is sometimes controversial what counts as a harm, and how significant it has to be for public policy to act to prevent it: a good example is the contemporary debate on second-hand smoke in public places[7]. Second, it can be easier to defend some restrictions on individual actions (stop smoking!), but harder to defend compelling people to do specific things (vaccinate your children!). The answer to this problem is that coercive legislation and other state interventions need good theoretical justifications and public, democratic oversight to ensure that they are both legitimate and proportionate to the threat being controlled. Good public health needs strong democracy[8,9].
